# Burst-Free Sustained Release of Proteins from Thermal Gelling Polymer Solutions

**DOI:** 10.3390/pharmaceutics17030376

**Published:** 2025-03-16

**Authors:** Yuxing Zhang, Xixi Zou, Qiran Du, Xiaotao Dong, Uday Kumar Chinta, Ruyue Yu, Fei Wu, Tuo Jin

**Affiliations:** School of Pharmacy, Shanghai Jiao Tong University, Shanghai 200240, China; seastar@sjtu.edu.cn (Y.Z.); xxzou_std@sjtu.edu.cn (X.Z.); clytie_du@sjtu.edu.cn (Q.D.); dongxiaotao@sjtu.edu.cn (X.D.); cuday91@sjtu.edu.cn (U.K.C.); yuruyue@sjtu.edu.cn (R.Y.)

**Keywords:** thermal gelling polymers, burst-free sustained release, dextran microparticles

## Abstract

**Objectives**: Thermo-gelling hydrophilic polymers like PLGA–PEG–PLGA are known as injectable sustained-release depots for biologics, but they face challenges due to the occurrence of severe burst release. This study aimed to develop a strategy to avoid the initial burst release by pre-encapsulating proteins in polysaccharide microparticles through an aqueous–aqueous emulsion mechanism, thereby enhancing therapeutic retention and linear release kinetics. **Methods**: Five model proteins (G-CSF, GM-CSF, IGF-1, FVIII, BSA) were encapsulated in dextran microparticles, using an organic solvent-free aqueous–aqueous emulsion method. These particles were dispersed in a 23% (*w*/*w*) PLGA–PEG–PLGA solution and injected into a 37 °C release buffer to form a gel depot. The in vitro release profiles were quantified using ELISA and MicroBCA assays over 9–42 days. The bioactivity of the proteins was validated using cell proliferation assays (NFS-60, TF-1, MCF-7) and chromogenic kits. The in vivo pharmacokinetics of the FVIII-loaded formulations were evaluated in Sprague–Dawley rats (n = 5/group) over 28 days. **Results**: Protein-loaded dextran particles retained their structural integrity within the hydrogel and exhibited minimal burst release (≤5% within 30 min vs. >25% for free proteins). Sustained near-linear release profiles were observed for all the proteins, with complete release by day 9 (G-CSF, GM-CSF, BSA) or day 42 (FVIII). Rats administered with the thermal gel with FVIII–dextran particles showed a significantly lower peak plasma concentration (C_max_: 88.25 ± 30.21 vs. 132.63 ± 66.67 ng/mL) and prolonged therapeutic coverage (>18 days vs. 15 days) compared to those administered with the thermal gel with the FVIII solution. The bioactivity of the released proteins remained at ≥90% of the native forms. **Conclusions**: Pre-encapsulation in dextran microparticles effectively mitigates burst release from thermosensitive hydrogels, while preserving protein functionality.

## 1. Introduction

Although the increasing number of tissue membrane-impermeable biologic medicines has created a great need for sustained-release systems to reduce injection frequency, practical dosage forms are currently limited to just a few products [1,2,3,4]. The essential difficulties in regard to developing sustained-release dosage forms for bio-drugs include a lack of reproducible production processes (for microspheres), severe burst release issues (for in situ thermo-gelling systems), and limited efficacious periods (for structurally modified proteins) [5,6,7,8,9,10,11,12,13,14]. The present study aims to resolve the burst release issue in terms of in situ thermo-gelling systems, using a handy formulation strategy. The selected PLGA–PEG–PLGA (PLGA, Poly(lactic-co-glycolic acid); PEG, polyethylene glycol) tri-block copolymer has been extensively characterized in regard to its biomedical compatibility, with its hydrolysis products (lactic acid, glycolic acid, and PEG) being naturally metabolized or excreted. Many previous studies, including clinical trials, have already confirmed its good biocompatibility by monitoring acute and chronic inflammation and through carrying out histopathological examinations of adjacent tissue [15,16,17,18]. This established safety profile positions it as an ideal candidate for injectable depots.

**Figure 1:**
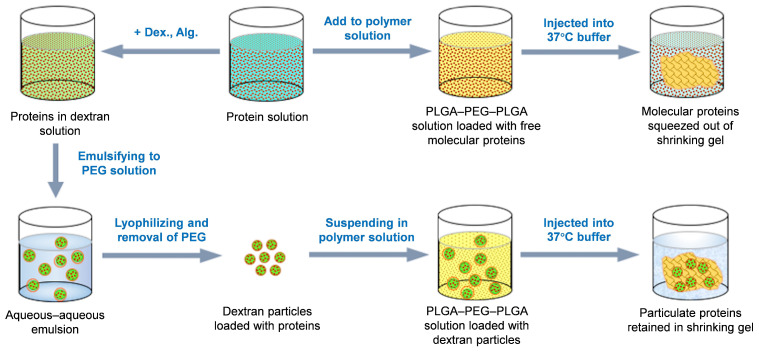
A schematic diagram of dextran particles in the PLGA–PEG–PLGA composite.

The mechanisms of burst release from thermo-gelling systems include the rapid diffusion of protein molecules before the gelling of the polymer solution at the injection site, and the extrusion of the protein solution by the gelling polymer due to the hydrophobic shrinking mechanism [19,20,21,22]. To reduce the initial burst, a unified approach is needed to simultaneously impede the two different molecular motions, thermal motion and mechanical extrusion. We hypothesize that pre-loading the protein safely in polysaccharide particles prior to mixing with the gelling polymer solution may meet this criterion. As schematically described in Figure 1, proteins may be encapsulated in polysaccharide particles thermodynamically. The encapsulation involved an organic solvent-free process, preserving the delicate structure of the proteins. These polysaccharide particles will neither dissolve nor release the proteins into the aqueous solution of the thermal gelling polymer, PLGA–PEG–PLGA, because its aqueous domains are PEG solutions. Polysaccharide solutions are immiscible with PEG solutions above a critical concentration, wherein preferential protein partitioning occurs during the polysaccharide phase [23,24,25]. When the PLGA–PEG–PLGA solution forms a gel through hydrophobic shrinking at an elevated temperature, the protein-retained polysaccharide particles that are several microns in diameter will be filtered out by the network of polymer chains from the extruding liquid [26] (Figure 1).

The practical significance of this hypothesized mechanism is that it may offer an extremely easy and effective solution for mitigating severe burst release, the long-standing hurdle blocking thermal gelling polymers from serving as a sustained-release dosage form of biological medicine. The feasibility of the polysaccharide particle-aided thermal gel system was confirmed experimentally in the present study using five proteins, the Granulocyte-colony stimulating factor (G-CSF), Granulocyte-macrophage colony stimulating factor (GM-CSF), Insulin-like growth factor (IGF-1), Human coagulation factor VIII (FVIII), and Bovine serum albumin (BSA), as model drugs.

## 2. Materials and Methods

### 2.1. Materials

Bovine serum albumin (BSA) was obtained from Si Ji, Ltd. (Shanghai, China). The Granulocyte-colony stimulating factor (G-CSF) was purchased from GenSci, Ltd. (Changchun, China). The Granulocyte-macrophage colony stimulating factor (GM-CSF) was purchased from Genova, Co. (Beijing, China). The Insulin-like growth factor (IGF-1) was purchased from GenScript, Ltd. (Nanjing, China). Human coagulation factor VIII (FVIII) was purchased from Anyuan Sciences, Ltd. (Shanghai, China). The G-CSF ELISA kit was purchased from Anyuan Sciences, Ltd. (Shanghai, China). The GM-CSF ELISA kit was purchased from Anyuan Sciences, Ltd. (Shanghai, China). The IGF-1 ELISA kit was purchased from MultiSciences (Lianke) Biotech, Co., Ltd. (Hangzhou, China). The FVIII ELISA kit was purchased from Signalway Antibody Ltd. (College Park, MD, USA). The FVIII activity chromogenic kit was purchased from Chromovenix, Ltd. (Bedford, MA, USA). Acetyl chloride was obtained from Aladdin, Ltd. (Shanghai, China). The BCA Protein Assay Kit was purchased from Pierce, Inc. (Appleton, WI, USA). Dichloromethane (CH_2_Cl_2_), Trichloromethane (CHCl_3_), Sodium Bicarbonate (NaHCO_3_), Calcium chloride anhydrous, Pyridine (C_5_H_5_N), sucrose, and Trehalose were obtained from Sinopharm Chemical Reagent, Ltd. (Shanghai, China). Polyethylene glycol (PEG, Mr = 1 kDa, 20 kDa), dextran (DEX, Mr = 64 kDa–76 kDa), sodium alginate (Alg, medium viscosity), stannous octoate, DL-Lactide, and glycolide were purchased from Sigma-Aldrich, Inc. (St. Louis, MO, USA). MCF-7, NFS-60, and TF-1 cells were purchased from the Cell Bank at the Chinese Academy of Sciences (Shanghai, China).

### 2.2. Animals

Male Sprague–Dawley (SD) rats (3–8 weeks, 200 g ± 50 g), purchased from Charles River Laboratory Co. Ltd. (Malvern, PA, USA), were used to study the in vivo release profiles. All the animals were kept in controlled temperature and humidity conditions. The study was approved by the institutional animal care and utilization committee at Shanghai Jiao Tong University, in compliance with the principles on the care of laboratory animals (Approval code: A2023195-003, 6 December 2023).

### 2.3. The Synthesis of the PLGA–PEG–PLGA Tri-Block Copolymer

The PLGA–PEG–PLGA tri-block copolymer (in-house synthesis) was synthesized using ring-opening polymerization, as previously reported [27,28,29,30,31,32,33]. In brief, polyethylene glycol (1 kDa), DL-Lactide, and glycolide (the molar ratios were 1:2.7:1) were mixed and allowed to react at 160 °C for 8 h, in the presence of stannous octoate as the initiator. Then, the reaction medium was cooled to room temperature and dissolved using CH_2_Cl_2_, followed by adding pyridine (5 equiv.). For purification, acetyl chloride was dropped into the reaction medium, under stirring at −10 °C for 5 h, followed by the evaporation of organic residues and dilution with cold water to about 6 °C. Finally, the target product was purified through precipitation at 80 °C and filtration.

### 2.4. Preparation of the Protein-Loaded DEX Particles

The model proteins, G-CSF, GM-CSF, IGF-1, FVIII, and BSA, were pre-loaded into dextran particles using the organic solvent-free process, aqueous–aqueous emulsification. The protein to be encapsulated was dissolved in a 20% (*w*/*w*) dextran solution and emulsified into a 20% (*w*/*w*) polyethylene glycol (PEG) solution, containing 1.5% (*w*/*w*) sodium alginate [34]. The molecular weight of the dextran was 70 kDa and that of PEG was above 20 kDa. A stable aqueous–aqueous emulsion was achieved by homogenizing the dextran/PEG mixture, using a JJ-1 electric homogenizer at 20,000 rpm for three consecutive 10 s intervals. The formed aqueous–aqueous emulsion was frozen at −20 °C overnight and lyophilized to a powder under a pressure of 5.25 × 10^−3^ Pa for 24 h, using an Alpha 1-2LD plus freezer dryer (ChristSigma Co., Ltd., Osterode am Harz, German). Finally, the lyophilized powder was suspended in dichloromethane or acetonitrile, followed by centrifugation at 14,000 r/min for 5 min, to remove the dissolved PEG. This washing procedure was repeated three times. Protein-loaded dextran particles were harvested after the washed powder was dried by evaporating the solvent residues.

### 2.5. Suspending Protein-Loaded Dextran Particles in the PLGA–PEG–PLGA Solution

The aqueous solution made of the thermal gelling PLGA–PEG–PLGA was prepared by blending the polymer in water at a concentration of 23% (*w*/*w*) at 4 °C. Then, the protein-loaded dextran particles were added into the polymer solution, with the mass ratio of dextran/PLGA–PEG–PLGA being 1:10 (*w*/*w*). The mixture was gently vortexed for 30 s and quickly warmed up to 37 °C in a water bath to form the thermal gel. For comparison, the same proteins in free-solution form were also added into the 23% (*w*/*w*) PLGA–PEG–PLGA solution, followed by quick warming up to 37 °C in a water bath.

The specifications of the thermal gel formulations of the examined model proteins are summarized in Table 1.

### 2.6. Determination of Phase Transition Temperature

To determine the temperature for the PLGA–PEG–PLGA solution, the sample was slowly warmed up in a water bath stepwise, at a rate of 1 °C/15 min, from 4 °C. The gelling state of the polymer solution was observed by inverting the sample vial 180 degrees for 30 s [7,35,36]. The temperatures of the samples when gelling and precipitation occurred were therefore identified.

### 2.7. Confocal Imaging of the Polymer Gelling Process

To control the temperature of the sample environment, we used a Nikon Ti-E live-cell fluorescence imaging system to take confocal images. Fluorescent BSA-loaded dextran particles were added into the PLGA–PEG–PLGA solution and vortexed for 30 s. One drop of the mixture was deposited on a glass slide and then placed in a cell culture incubator set at 10 °C. After taking images at 10 °C, the system temperature was elevated to 37 °C to induce polymer gelation. The images were taken under the same conditions and with the same field of view.

### 2.8. Protein Activity Assessment Throughout the Formulation Process and In Vitro Release

The preservation of the activities of the bio-active proteins, G-CSF, GM-CSF, and IGF-1, throughout the formulation process was assayed using the proliferation rate of the NFS-60, TF-1, and MCF-7 cells, respectively. The bioactivity of FVIII was assayed using the FVIII activity chromogenic kit. These assays were carried out after each formulation step, as well as for each sampling point during the in vitro release.

### 2.9. In Vitro Release Profile Assays for the Proteins from the PLGA–PEG–PLGA Thermal Gel Formulations

The release profiles of the model proteins, G-CSF, GM-CSF, IGF-1 FVIII, and BSA, from the thermal gel formulations F1 to F9, as shown in Table 1, were examined by adding the sample into a release vial and heating in a water bath at 37 °C. After gel formation, a release buffer was added into the vial. The release medium was collected, followed by re-filling with the same amount of fresh medium, at each sampling time of 0.5 h, 1, 2, 3, 4, 5, 6, 7, 9, 11, and 13 days for G-CSF, GM-CSF, and BSA; at each sampling time of 1, 2, 3, 4, 5, 6, 7, 9, 11, and 13 days for IGF-1; and at each sampling time of 1, 2, 3, 4, 5, 6, 7, 8, 9, 10, 11, 12, 13, 14, 15, 16, 17, 18, 20, 22, 24, 28, 35, and 42 days for FVIII. The released protein amounts were determined using ELISA (for G-CSF, GM-CSF, IGF-1, and FVIII) and MicroBCA (for BSA) assays, respectively. The cumulative release amount of the proteins was plotted against the sampling time to form release profile curves. The bioactivity of the released G-CSF, GM-CSF, IGF-1, and FVIII were assayed for comparison with the release amount (refer to Section 2.8).

### 2.10. In Vivo Release Profiles After Subcutaneous Injection of the PLGA–PEG–PLGA Thermal Gel Formulation

We chose male SD rats as the animal model to evaluate the in vivo performance of the thermal gel formulation, and the rats were divided into two groups (n = 5). A total of 500 μL of the PLGA–PEG–PLGA thermal gel mixed FVIII solution or dextran particles was injected subcutaneously into the abdomen of the rats in each group, respectively, and the dose of FVIII was 420 μg/kg. At a time of 1 h, 1, 2, 3, 4, 5, 6, 8, 10, 12, 15, 18, 21, 24, and 28 days after administration, approximately 0.5 mL blood was collected from the retro-orbital sinus of each rat and treated with EDTA-K. The blood samples were centrifuged at 3000× *g* at 4 °C within 2 h to separate the plasma and were stored at −40 °C before the analysis. The plasma concentration of FVIII was detected using an ELISA kit, and the rat plasma was used as the standard diluent to minimize any interference.

## 3. Results and Discussion

### 3.1. Insolubility of Polysaccharide Particles in the Aqueous PLGA–PEG–PLGA Solution

**Figure 2:**
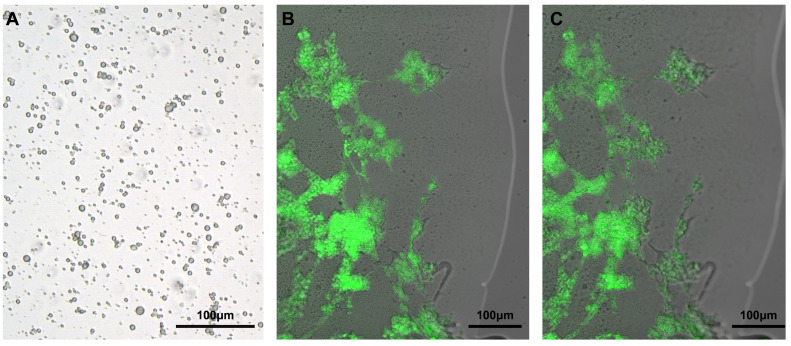
(**A**) Optical microscopic image of BSA–DEX particles using aqueous–aqueous emulsification; confocal microscopic images of fluorescent BSA-loaded dextran particle retention in the PLGA–PEG–PLGA thermosensitive hydrogel at different temperatures: (**B**) 10 °C and (**C**) 37 °C.

The premise of this pre-formulation strategy is that the protein-loaded polysaccharide particles do not dissolve in the aqueous domain of the thermal gelling polymer solution. This hypothesis was confirmed by suspending dextran particles, which were loaded with bovine serum albumin (BSA), in the PLGA–PEG–PLGA solution, followed by microscopic observation. As indicated in Figure 2, the BSA-loaded dextran particles made through aqueous–aqueous emulsion method retained their shape completely when suspended in the polymer solution. The structural integrity of the dextran particles within the PLGA–PEG–PLGA hydrogel is maintained through purely physical dispersion rather than chemical interactions. Dextran exhibits no electrostatic attraction or hydrophobic force toward the PLGA–PEG–PLGA polymer, allowing uniform distribution within the hydrophilic PEG domains through gentle mixing.

### 3.2. Retention of Polysaccharide Particles During the Polymer Gelling Process

Warming up the 23% (*w*/*w*) aqueous PLGA–PEG–PLGA solution in a water bath to 30 °C resulted in quick gelling, indicating that some of the hydrophobic fragments of the polymer aggregated to form hydrophobic domains, functioning as crosslinking junctions. A further increase in the temperature of the water bath to 37 °C led to the separation of the PLGA–PEG–PLGA system to a size-reduced gel phase and free-water phase, suggesting the occurrence of hydrophobic shrinkage during the polymer phase due to further dehydration. The shrunk polymer gel at 37 °C appeared to be significantly firmer than that at 30 °C when touched with a glass rod, suggesting that the gel may form a sustained release depot at the injection site. To examine whether particles may be retained inside the polymer network during the gelling process, we suspended fluorescent BSA-loaded dextran particles in the PLGA–PEG–PLGA solution and took photos of this system at both 10 °C (solution state) and 37 °C (gel state). The confocal microscopic images confirmed that the BSA-loaded dextran particles were retained intact in the matrix of the gel (Figure 2B,C), consistent with our initial hypothesis.

### 3.3. Preservation of Protein Activity During the Formulation Processes and Release Process

**Figure 3:**
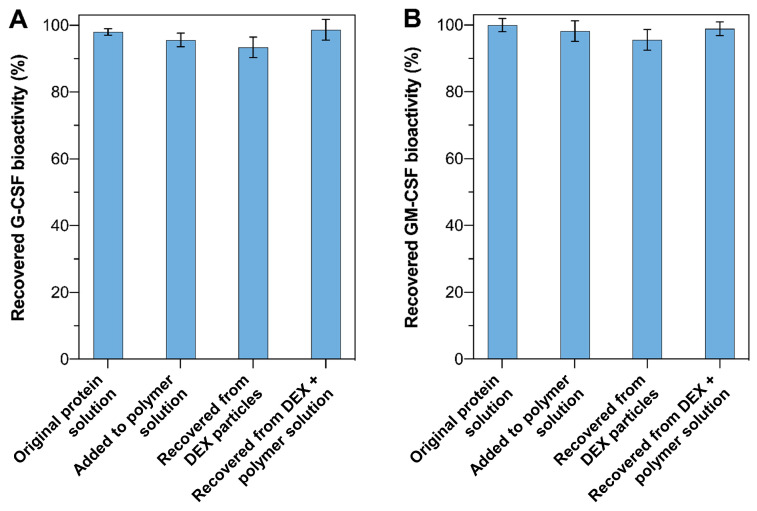
Activity of different recovered proteins after formulation steps: (**A**) G-CSF and (**B**) GM-CSF; (n = 3).

The friendliness of this formulation process in preserving the native state of protein drugs was confirmed by the activity assessment following each of the formulation steps. The two model protein drugs, G-CSF and GM-CSF, were recovered from the dextran particles prepared via the aqueous–aqueous emulsification method and from the PLGA–PEG–PLGA copolymer gel loaded with the particles. The recovered proteins, as well as the proteins added directly into the polymer solution, were diluted to match the concentration of the original diluted protein solution, and tested in regard to their activity to stimulate the proliferation of NFS-60 and TF-1 cells, they were then compared with the original protein solutions, with the same concentration. As summarized in Figure 3, the G-CSF and GM-CSF recovered from each formulation step showed no significant difference in regard to the cell proliferation activity as compared with the original protein solutions.

**Figure 4:**
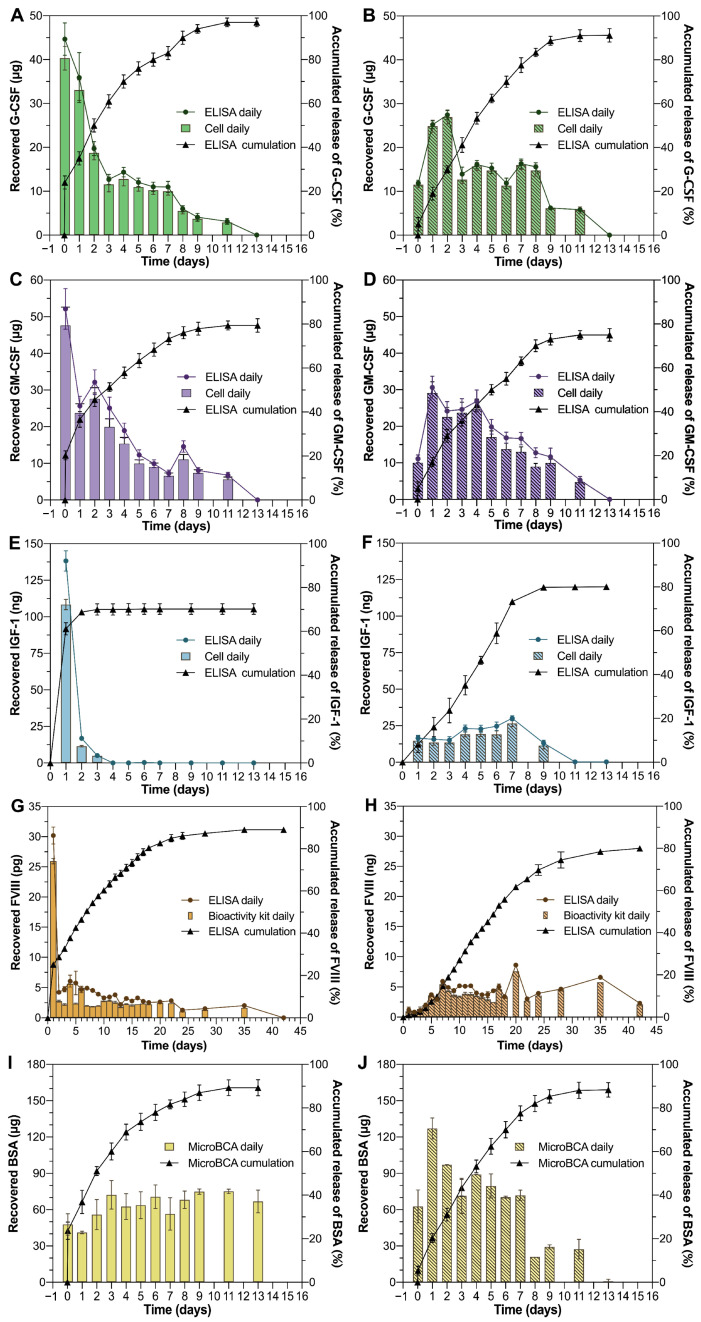
Bioactivity and release profile of different proteins: (**A**,**C**,**E**,**G**,**I**) proteins added directly into the polymer solution; (**B**,**D**,**F**,**H**,**J**) proteins recovered from DEX–ALG particles in the polymer solution; (**A**,**B**) G-CSF; (**C**,**D**) GM-CSF; (**E**,**F**) IGF-1; (**G**,**H**) FVIII; (**I**,**J**) BSA; (n = 3).

The bioactivity of the proteins recovered during the release process is shown in Figure 4. When compared to those recovered from the polymer solution without dextran particles, the proteins recovered from the dextran particles in the polymer solution throughout the release process showed higher bioactivity. The level of protein activity preservation observed in the present study is consistent with the findings reported in many studies for PLGA–PEG–PLGA thermal gel systems [37,38,39,40].

### 3.4. In Vitro Release Kinetics of Proteins from Thermal Gelling PLGA–PEG–PLGA

The main goal of this study is to confirm the feasibility of our proposed strategy to achieve burst-free sustained release of proteins from the PLGA–PEG–PLGA thermal gel depot, one of the few polymeric materials accepted as an injectable dosage form in this category. The strategy employed was to pre-load the proteins in polysaccharide particles and retain the particles in the network of the shrinking copolymer depot, which would eventually result in reduced burst release. The capability of the protein particle-loaded thermal gel system to achieve this goal was examined by injecting the gel-forming PLGA–PEG–PLGA solution into a release medium at 37 °C, followed by assaying the amount of proteins released. The results were, as indicated in Figure 4, surprisingly optimistic. For all the three model protein drugs, G-CSF, GM-CSF, and BSA, the protein release in the initial 30 min was below 5% of the total drug loading from the dextran particle-aided thermal gel, while the release of proteins from the same gelling polymer without dextran particle retention was over 25%. However, for IGF-1, during the first day, the release of the protein loaded in the dextran particle-aided thermal gel was about 8%, but the release of the proteins added directly into the same gelling polymer was over 60%. Furthermore, for FVIII, there was no obvious burst release of the protein from the dextran particle-aided thermal gel during the first day (below 1%), while the release of the protein from the same gelling solution without dextran particles was over 20%. Clearly, the dextran particles neither went with the extruded solution by themselves, nor did they quickly release the pre-loaded proteins into the hydrophilic PEG domain of the G-CSF, GM-CSF, IGF-1, and FVIII depot. The partitioning of the proteins favoring the polysaccharide phase, rather than the PEG phase, played an essential role in this process. In regard to the dextran particles prepared using the two different methods, however, no significant differences were identified in the protein release kinetics.

It is also interesting that all the proteins showed nearly linear release profiles from the particle-aided thermal gel depots until the depletion of the drug content. The proteins, including G-CSF, GM-CSF IGF-1, and BSA, were almost completely released by day 9 of the experiment, while the release of FVIII lasted more than 28 days (Figure 4). This release profile must not be attributed merely to the diffusion of the macromolecules, but an additional mechanism that gradually accelerates the protein release to compensate for the reduced rate of diffusion. We speculate that the dissociation of the dextran particles, due to the gradual hydration of the degrading depot, played a role in accelerating the protein release, wherein the proteins could no longer be partitioned preferentially during each separated dextran phase. An immiscible aqueous two-phase system, such as dextran and PEG solutions, becomes miscible when the polymer solutions are diluted.

### 3.5. In Vivo Release Performance of FVIII from Thermal Gelling PLGA–PEG–PLGA

**Figure 5:**
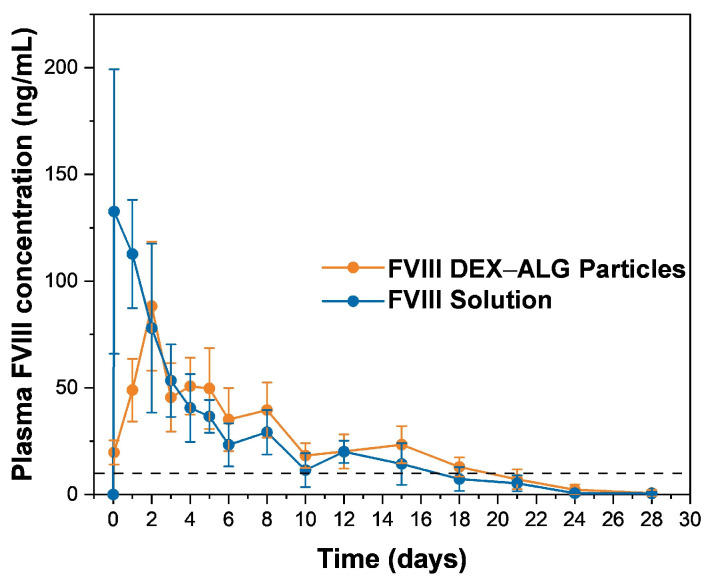
In vivo release profile of FVIII DEX–ALG particles and FVIII solution. (The dotted line refers to the minimum therapeutic concentration in plasma.); (n = 5).

To verify the effect of dextran particles in vivo, we used FVIII as a model protein to complement further animal experiments, given the requirement for sustained-release FVIII formulations for the treatment of hemophilia A patients. The results, which are shown in Figure 5, show that the rats injected with the thermal gel mixed with the FVIII solution (group 1) experienced a burst release at 1 h after administration and reached a peak plasma concentration of 132.63 ± 66.67 ng/mL at once. In contrast, the group given the dextran particles (group 2) reached a C_max_ of 88.25 ± 30.21 ng/mL during day 2 after administration, which was consistent with the milder initial release observed in the in vitro experiment. Apparently, group 2 exhibited prolonged sustained performance, with the plasma concentration remaining above the minimum therapeutically effective concentration (10 ng/mL, indicated by the dotted line in Figure 5) [41] until 18 days after administration, while in group 1, the plasma concentration level was maintained above the minimum therapeutically effective concentration for 15 days and decreased to a normal level subsequently. Based on the in vivo profiles, we calculated the AUC_1–28_ of both groups, namely 515.04 ± 92.91 ng·days/mL^−1^ for group 1 and 617.12 ± 109.54 ng·days/mL^−1^ for group 2. It is encouraging that the AUC of group 2 was higher than group 1, which indicated a prolonged effect after the inhibition of the burst release of the proteins. The in vivo release performance proved the feasibility of dextran particles for in situ injection of thermal gels subcutaneously, as they reduced the early burst release of the proteins from the thermal gel due to their advantageous micro-size. However, the whole in vivo release process did not last as long as the 42 days observed in terms of the in vitro release process. Two factors may contribute to the duration discrepancy. In regard to the in vitro model, PLGA–PEG–PLGA was placed at the bottom of the vial, so that a limited surface area was exposed to the release medium. Subcutaneous administration in vivo enables 360° surface contact with physiological fluids, resulting in accelerated gel degradation and protein diffusion. Furthermore, as a macromolecular with a high molecular weight (~280 kDa), FVIII enters the systemic circulation through lymphatic drainage rather than direct capillary absorption. During this process, some FVIII was inactivated due to proteolytic degradation after binding to phospholipids, resulting in reduced bioavailability [42].

### 3.6. Feasibility of the Particle-Aided Thermal Gelling Polymer Depot as a Practical Dosage Form

The most encouraging advantage of pre-loading proteins into polysaccharide particles before gel formulation is that it effectively resolved the long-standing burst release problem in in situ thermal gels without the need for extensive exploration of new polymers. The thermal gelling copolymer used in the present study, PLGA–PEG–PLGA, is approved for injection in the healthcare industry. Moreover, pre-loading delicate proteins into dextran particles is easy to operate and involves a process that favors thermodynamics that avoids exposing macromolecules to water–oil or water–air interfaces, which are known to denature proteins. It may seem awkward that most of the proteins’ in vitro release lasted for only 9 days, slightly shorter than the ideal two-week period for convenient and easy-to-remember drug administration. However, the in vitro and in vivo performance of FVIII may inspire us regarding the tremendous potential in regard to the application of proteins with a high molecular weight, which exhibit promising in vitro release profiles, lasting for several weeks. Moreover, optimizing the molecular weight of PLGA–PEG–PLGA and dextran would also extend the protein release process.

While the current study focuses on resolving the burst-release challenge, the potential clinical translation of this solution requires further consideration. The first aspect is formulation stability. As lyophilization is a well-established stabilization technique, we believe the PLGA–PEG–PLGA tri-block copolymer and dextran particles can be stabilized in dry conditions for a long time. But this still needs further investigation. Meanwhile, the solvent-free process aligns with green chemistry principles, and the standard unit operations (homogenization, lyophilization) used in this study are directly scalable in regard to GMP production, indicating their potential for translation to clinical practice.

## 4. Conclusions

In conclusion, this study establishes a sustained-release delivery platform for proteins through the innovative combination of solvent-free aqueous–aqueous encapsulation, using thermal hydrogel technology. Without using organic solvents, we achieve >90% bioactivity preservation during the preparation process. The platform’s broad applicability is demonstrated by the successful encapsulation and sustained release of proteins ranging from 6.5 kDa (G-CSF) to 280 kDa (FVIII), eliminating the burst release problem for thermo-gelling systems. The near-linear release profiles lasted more than 9 days, and this trend was consistent in vivo, exhibiting lower C_max_ values and an extended sustained release period.

In the future, this thermo-sensitive hydrogel can be exploited for wound healing medications, skincare products, eye drops, and other applications. Overall, pre-loading proteins in polysaccharide particles through the use of an all-aqueous phase process prior to gel formulation may offer an immediate solution to achieve practically feasible sustained-release dosage forms of delicate protein drugs.

## Figures and Tables

**Table 1:** Contents and formulation methods for the protein-loaded thermal gelling PLGA–PEG–PLGA solution.

No.	Formulation	Composition (*w*/*w*)	Method for Preparing DEX Particles
F1	GM-CSF, PLGA–PEG–PLGA	1/60	/
F2	GM-CSF, DEX, ALG, PLGA–PEG–PLGA	1/5/1.875/78.75	double aqueous emulsion
F3	G-CSF, sucrose, PLGA–PEG–PLGA	1/1/70	/
F4	G-CSF, sucrose, DEX, ALG, PLGA–PEG–PLGA	1/1/5/1.875/88.75	double aqueous emulsion
F5	IGF-1, PLGA–PEG–PLGA	1/6187.5	/
F6	IGF-1, DEX, ALG, PLGA–PEG–PLGA	1/500/187.5/6187.5	double aqueous emulsion
F7	FVIII, PLGA–PEG–PLGA	1/132.75	/
F8	FVIII, DEX, ALG, PLGA–PEG–PLGA	1/10/3.75/132.75	double aqueous emulsion
F9	BSA, PLGA–PEG–PLGA	1/60	/
F10	BSA, DEX, ALG, PLGA–PEG–PLGA	1/5/1.875/78.75	double aqueous emulsion

## Data Availability

Data is contained within the article.

## Abbreviations

The following abbreviations are used in this manuscript:

PEG	Polyethylene glycol
PLGA	Poly(lactic-co-glycolic acid)
DEX	Dextran
ALG	Sodium alginate
G-CSF	Granulocyte-colony stimulating factor
GM-CSF	Granulocyte-macrophage colony stimulating factor
BSA	Bovine serum albumin
IGF	Insulin-like growth factor
